# Supplementary material for: Health professionals’ readiness and its associated factors to implement Telemedicine system at private hospitals in Amhara region, Ethiopia 2021
Source: PLoS One. 2022 Sep 29;17(9):e0275133. doi: 10.1371/journal.pone.0275133 (PMC9522275; doi:10.1371/journal.pone.0275133)
Supplement: Attachment — Submitted filename: Rebuttal letter.docx [file pone.0275133.s002.docx]

**Dear Editors of PLOS ONE :**

It has been recalled that we the authors of the manuscript entitled “**Health Professionals' readiness and its associated factors to implement Telemedicine system at private Hospitals in Amhara Region, Ethiopia 2021**” submitted our manuscript for publication in your journal and received reviewer comments for the betterment of the manuscript before its publication. In line with this, all authors are very happy with the constructive and valuable comments given by reviewers. Accordingly, we have considered all the comments and provided a point-by-point response and explanations for all the questions raised. Finally, we have submitted all the required documents in their revised form. We hope that we have addressed all the questions and if you have any points for further clarity, let us know.

All the authors would like to thank the editorial team and reviewers

**Editor(s)’ comments to the authors**

**Comment1:** A rebuttal letter that responds to each point raised by the academic editor and reviewer(s). You should upload this letter as a separate file labeled 'Response to Reviewers'.

**Answer:** Thanks dear editor for your nice comments and suggestions. We, the authors
of this study, have attached the necessary files and a detailed rebuttal letter according to
your suggestion and the journal format.

**Comment2:** A marked-up copy of your manuscript that highlights changes made to the original version. You should upload this as a separate file labeled 'Revised Manuscript with Track Changes.

**Answer:** Thank you, the track changes and cleaned document have been prepared and labeled as revised manuscript and attached

**Comment 3:** An unmarked version of your revised paper without tracked changes. You should upload this as a separate file labeled 'Manuscript'.

**Answer:** thank you very much, dear editor, unmarked version of the revised manuscript was prepared without track change labeled as the manuscript was uploaded.

**Journal Requirements:**

**Comments:** Please review your reference list to ensure that it is complete and correct. If you have cited papers that have been retracted, please include the rationale for doing so in the manuscript text, or remove these references and replace them with relevant current references. Any changes to the reference list should be mentioned in the rebuttal letter that accompanies your revised manuscript. If you need to cite a retracted article, indicate the article’s retracted status in the References list and also include a citation and full reference for the retraction notice.

**Answer:** thank you dear editor we authors of this study agreed with your comments and suggestions. We tried to review the whole references cited in the manuscript are complete and correct. we ensured that all references are correct and complete no retracted papers are cited in our manuscript.

**Reviewer 1 comment to the author**

**Comment#reviwer1:** In conclusion, the macro view should be observed and the impact of this research on the successful use of telemedicine and the improvement of the health system should be mentioned.

**Answer:** thank you very much for your valuable comments ad suggestion for the improvement of our manuscript, we authors are happy to accept and agreed with the comments. Accordingly, we revised the conclusion section as recommended in the main revised manuscript.

**Comment#reviwer1:** To improve the validity of the sentence "In the era of covid-19 pandemics telemedicine have a great role in controlling the pandemics by using different communication …." in the introduction section (lines 66-69) on page 15, refer to the following reference.

**Answer:** thank you very much, dear reviewer, for your insightful comments and suggestion for our manuscript development. We authors of this study gladly accepted and agreed with your comments. We tried to cite the recommended reference in the introduction section lines66-69 in the revised manuscript.

**Reviwer2 comments to the Author**

Reviewer #2: The authors have adequately addressed my comments raised in a previous round of review and I believe that this manuscript is now acceptable for publication,

**Answer:** thank you very much dear reviewer for your insightful appreciation and recognition, we authors of this study gladly accepted and admiration for your constructive scientific comments forwarded to our manuscript improvement. Thank you very much again.
